# ARG-based genome-wide analysis of cacao cultivars

**DOI:** 10.1186/1471-2105-13-S19-S17

**Published:** 2012-12-19

**Authors:** Filippo Utro, Omar Eduardo Cornejo, Donald Livingstone, Juan Carlos Motamayor, Laxmi Parida

**Affiliations:** 1 Computational Biology Center, IBM T. J. Watson Research, Yorktown Heights, NY 10598, USA; 2 Department of Genetics, Stanford University School of Medicine, Stanford, CA 94305-5120, USA; 3 USDA, Miami, FL 33186, USA; 4 Mars Inc., Miami, FL 33158, USA

## Abstract

**Background:**

Ancestral recombinations graph (ARG) is a topological structure that captures the relationship between the extant genomic sequences in terms of genetic events including recombinations. IRiS is a system that estimates the ARG on sequences of individuals, at genomic scales, capturing the relationship between these individuals of the species. Recently, this system was used to estimate the ARG of the recombining X Chromosome of a collection of human populations using relatively dense, bi-allelic SNP data.

**Results:**

While the ARG is a natural model for capturing the inter-relationship between a single chromosome of the individuals of a species, it is not immediately apparent how the model can utilize whole-genome (across chromosomes) diploid data. Also, the sheer complexity of an ARG structure presents a challenge to graph visualization techniques. In this paper we examine the ARG reconstruction for (1) genome-wide or multiple chromosomes, (2) multi-allelic and (3) extremely sparse data. To aid in the visualization of the results of the reconstructed ARG, we additionally construct a much simplified topology, a classification tree, suggested by the ARG.

As the test case, we study the problem of extracting the relationship between populations of *Theobroma cacao*. The chocolate tree is an outcrossing species in the wild, due to self-incompatibility mechanisms at play. Thus a principled approach to understanding the inter-relationships between the different populations must take the shuffling of the genomic segments into account. The polymorphisms in the test data are short tandem repeats (STR) and are multi-allelic (sometimes as high as 30 distinct possible values at a locus). Each is at a genomic location that is bilaterally transmitted, hence the ARG is a natural model for this data. Another characteristic of this plant data set is that while it is genome-wide, across 10 linkage groups or chromosomes, it is very sparse, i.e., only 96 loci from a genome of approximately 400 megabases. The results are visualized both as MDS plots and as classification trees. To evaluate the accuracy of the ARG approach, we compare the results with those available in literature.

**Conclusions:**

We have extended the ARG model to incorporate genome-wide (ensemble of multiple chromosomes) data in a natural way. We present a simple scheme to implement this in practice. Finally, this is the first time that a plant population data set is being studied by estimating its underlying ARG. We demonstrate an overall precision of 0.92 and an overall recall of 0.93 of the ARG-based classification, with respect to the gold standard. While we have corroborated the classification of the samples with that in literature, this opens the door to other potential studies that can be made on the ARG.

## Background

In this paper we apply an interesting population genomics tool to an equally fascinating plant. While the consumption of chocolate is very high in Europe and North America, interestingly, Cacao provides a livelihood for over six million farmers in Africa, South America and Asia. It ranks as one of the top ten agriculture commodities in the world. An understanding of the classification is important since breeders capitalize on the heterosis between distinct genetic groups to increase yield. The cacao's mating system renders cultivars to be self-compatible while the varieties in the wild are not. Thus understanding the genetic relationship between the varieties is a problem of great interest. To give a historical perspective, the problem of classification of Cacao, based on morphological data, has been difficult since the evolution of the cacao diversity took place over the entire primary distribution area in the Amazonian region and was also affected by the arrival of the Europeans into the Amazon region [1]. However, more recently, molecular markers have been used to classify cacao varieties [2-4]. The most thorough classification is provided by Motamayor et al. [5] where the authors suggest 10 genetic groups based on genome-wide STR data. We use this data set to apply the ARG approach to test it against their classification.

A principled approach to understanding the inter-relationships between populations must take the shuffling of the genomic segments into account. IRiS studies the different samples from different populations/varieties by constructing an underlying ARG, at genomic scales [6]. Here we use the data set that is used in [5] to obtain an ARG-based classification, in 10 genetic groups, of about a thousand cacao samples, each with about a hundred STR polymorphic loci, originally collected from different geographic regions in the Americas. In this paper, we extend the IRiS model to handle this sparse, multi-allelic data over multiple chromosomes.

The advantage of the ARG approach is that it attempts to precisely model the underlying genetic events, the most plausible reasons offered by biology, to explain parsimoniously the observed genetic differences in the samples. This level of directness -of cause and effect- in the approach is quite appealing but the complexity of the resulting ARG is quite often overwhelming. Hence, we summarize the ARG as classification trees for both easy consumption as well as comparison with other known results. We demonstrate an overall precision of 0.92 and an overall recall of 0.93 of the ARG-based clusters, with respect to the classification suggested in [5].

## Methods

The germplasm samples are derived from twelve different American countries: Belize, Brazil, Colombia, Costa Rica, Ecuador, French Guiana, Ghana, Mexico, Nicaragua, Panama, Perú and Venezuela. After accounting for inconsistencies and other errors, 952 samples, each with 96 STR polymorphisms across the 10 chromosomes are subject to the classification process via computational methods [5]. The distribution of the polymorphic loci is not uniform and only half the chromosomes have about ten STR loci. The data is *n*-allelic (as opposed to bi-allelic) with number of distinct values at a locus varying from 3 to 30.

**Figure 1:**
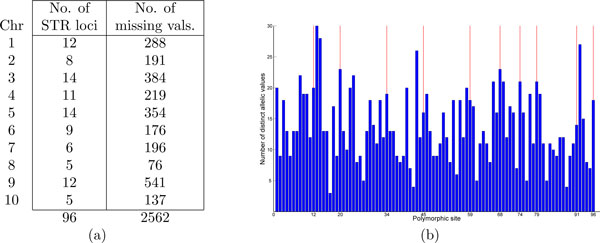
**The essential characteristics of the data set**. (a) A summary of the distribution of the polymorphic sites over the chromosomes and the missing values. (b) Number of distinct allelic values per polymorphic site (which ranges from 3 to 30). The red vertical lines are the boundaries of the chromosomes.

Additionally, a total of 2562 values are missing, spread somewhat randomly through the data. Since the data set is quite sparse (i.e. only 96 markers genome-wide), we use a simple approach to impute the missing values: we estimate the missing values by using the most common haplotype seen across the 952 samples, breaking ties arbitrarily. The essential characteristics of this data set, from an analysis perspective, is summarized in Figure 1. Notice the large number of distinct alleles at most of the loci. However, the data is rather complete with only about 3% missing values. We also noticed that results did not vary much when no imputation was carried out (with the missing values being assigned an arbitrary distinct value). However, all the results reported here have used the imputed version of the data set. Additionally, in [5] a careful subsample of only 559 (out of the 952) have been used to construct the classification tree. We have also used this subsample to test the efficacy of both the subsampling and that of our methods.

### Can a classification be done on the raw STR data?

Is it possible for a framework, that does not model evolution in any form, to discern structure in this data set? To answer this question we first tried various classical clustering algorithms on the data at hand. These clustering algorithms are not aware of the data domain and simply work on the observed values. We selected a set of five clustering algorithms for this experiment. For methods that required the number of clusters, we tried values from seven to fifteen. The methods are: Average Linkage, Complete Linkage, Single Linkage and *K*-means with random initialization [7]. The classification methods are dominated by hierarchical clustering algorithms and this is also reflected in our choice of the algorithms. Note that the first three are hierarchical clustering algorithms. We also used the nearest neighbor joining (NNJ) algorithm which has been frequently used on genomic data. For completeness, we describe the methods in broad strokes below and the interested reader is directed to [7-10] for details.

The hierarchical clustering algorithms produces a partition by a nested sequence of partitions which give a natural classification tree. These algorithms can be either agglomerative (bottom-up), in which one starts at the leaves and successively merges clusters together; or divisive (top-down) in which one starts at the root and recursively splits the clusters. NNJ is a bottom-up hierarchical clustering method, based on the minimum-evolution criterion, for the creation of phenetic trees. *K*-means clustering algorithms decompose the data set directly into a set of disjoint clusters. Intuitively, the aim is to minimize the dissimilarity between items in the same cluster and to maximize the dissimilarity between items of different clusters.

These general methods were used in conjunction with classical distance/similiarity measures (i.e. Euclidean). They gave poor results such as a single large cluster and many small, including singleton, clusters. We do not report these results here, since no consistent classifications could be extracted from them. The interested reader can find the relevant results in Additional File 1. We conclude that most of the methods used on the raw STR data, does not yield robust classifications of the cacao samples.

### An ARG-based approach

Recall that an ARG is a topological structure that captures the relationship between the extant genomic sequences in terms of genetic events including recombinations. Graphically, it is a directed acyclic graph where each node corresponds to a unit, at some generation, and the edges denote the transmission of genetic materials between the units. The extant units are at the leaf level, i.e., they have no outgoing edges. The nodes can have single incoming edges or multiple incoming edges. The former is called a *coalescent *node and the latter a *genetic exchange *node. The reader is directed to [11] for details. The ARG is defined over the same locus for multiple given samples or extant units and IRiS is a system that reconstructs this ARG given the sequences/markers on the samples [6].

In principle, an ARG can be constructed only on a segment or the whole chromosome. Since the data at hand is very poor in terms of density, we employ two methods to work across multiple chromosomes. In both the methods, the analysis is done in two stages. At the first stage, the chromosomes are presented in some coherent fashion to the IRiS pipeline. This is iterated multiple times. In the second stage, the results from the multiple iterations are consolidated.

For both the methods we use the complete (952) data set and the subsample (559) data. Since the subsample has been carefully curated, we check the robustness of this curation using the ARG approach.

#### 1. The Solo Method

In this method, some *Z' *chromosomes are picked and then staged separately to the IRiS pipeline. In our experiments we use (i) the first three longest chromosomes and (ii) all ten chromosomes.

While this is the most natural mode of using the ARG approach, it appears that there is more information in the multiple chromosomes that is not exploited here. Since the data is so sparse (only about a hundred loci genome-wide), it is important to use all possible signatures. The intra-chromosome commonalities are captured by the use of the chromosome for the ARG. The following method aims to additionally capture the inter-chromosome commonalities.

#### 2. The Ensemble Method

**Figure 2:**
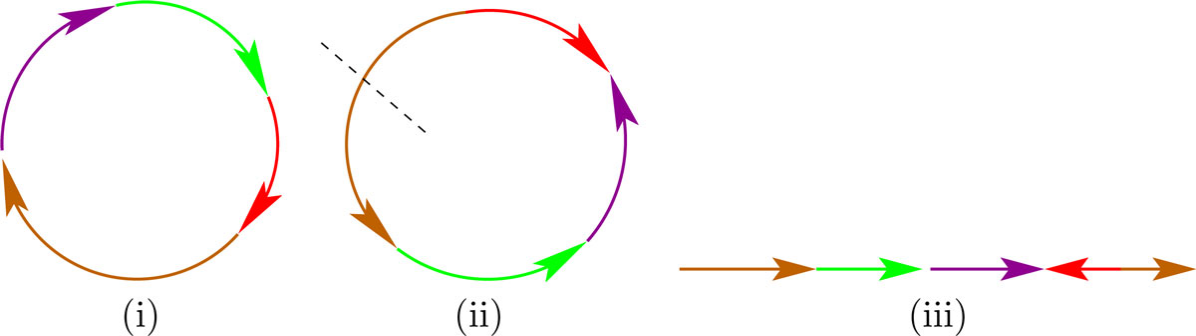
**The Ensemble Method**. The Ensemble Method: An example with 4 chromosomes each with an orientation and a distinct color above. (i) The chromosomes are first arranged as a circular ring. (ii) Then they are randomly permuted and randomly flipped. Then a random cut is placed on the ring, shown as a dashed line. (iii) Then the ring is flattened out and is staged as input to the IRiS pipeline.

Here *G^'^*chromosomes are used to produce a single sequence that is staged as input to the IRiS pipeline. This single *ensemble *is produced from the *G^' ^*chromosomes in the following three steps (see Figure 2). First, the *G' *chromosomes are placed in some random order in the 5' to 3' order in a circle. Then a random subset of these are flipped (to be in 3' to 5' order). Finally, the circle is cut by introducing a break at a random position. Then this linear string is staged as input to the IRiS pipeline. This is repeated some *N *times and the results are consolidated.

As discussed before, the rationale for the ensemble method is that, indeed the signature generated by multiple chromosomes following a single chromosome or *vice-versa*, does capture essential commonalities across individuals. The same can be said of flipping the direction of each individual chromosome. Indeed, the best results are seen in the Ensemble method, as discussed later.

#### Consolidation of the multiple iterations

**Figure 3:**
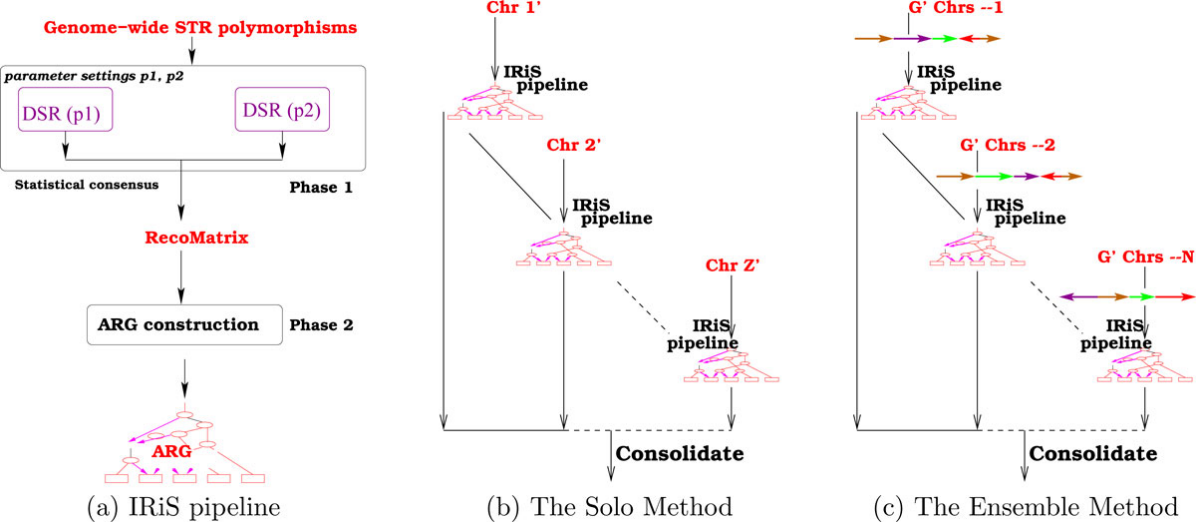
**The work flow for multiple chromosomes**. Work flow for multiple (*G*') chromosomes. (a) IRiS pipeline: In Phase 1, we use two different parameter settings to obtain an intermediate matrix (called recomatrix) for Phase 2. The second phase constructs the ARG. (b) Some Z' ≤ *G' *chromosomes are used through the IRiS pipeline and the *Z' *results are consolidated to obtain the final analysis. (c) The IRiS pipeline is used on an ensemble sequence (see Fig. 2) of the *G' *chromosomes some *N *times and the results are consolidated.

**Figure 4:**
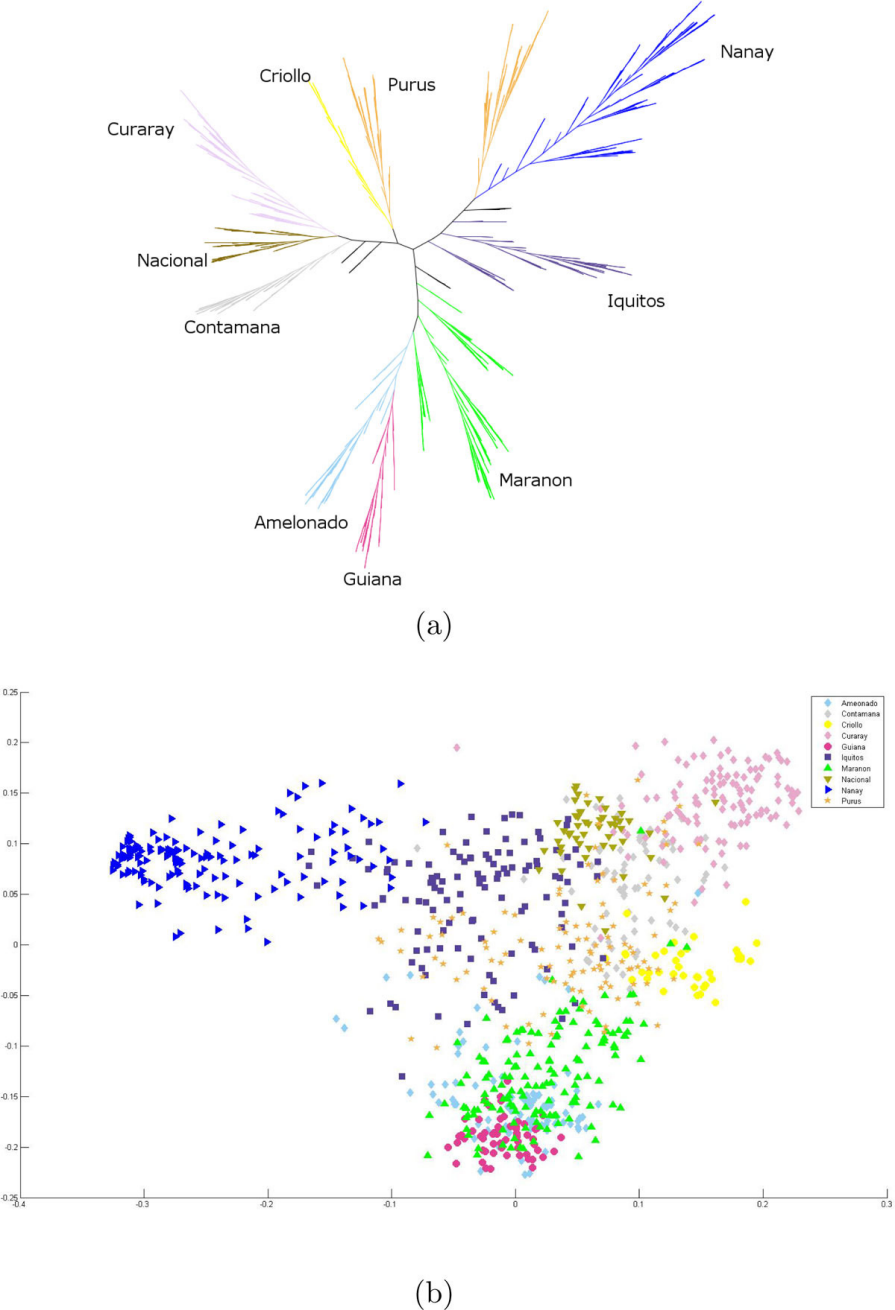
**Results for the ensemble method on the complete data set**. Visualization of the ARG results as a classification tree: The Ensemble method on all ten chromosomes on the complete data set. (a) A classification tree. (b) The first two components of an MDS of the pairwise distances.

**Figure 5:**
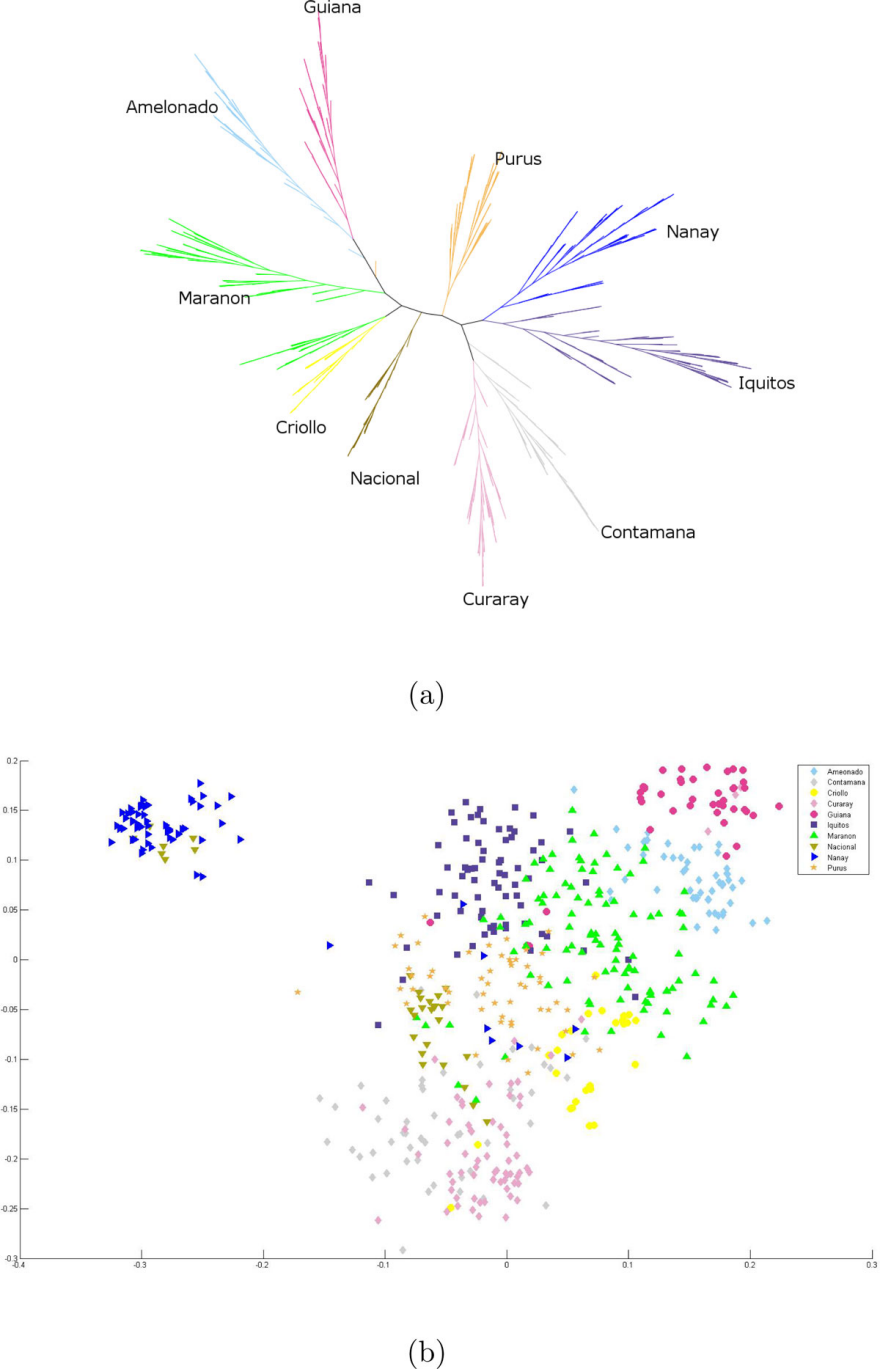
**Results for the ensemble method on the subsample data set**. Visualization of the ARG results as a classification tree and an MDS plot: The Ensemble method on all ten chromosomes on the subsample data set. (a) A classification tree. Note that [5] presents a classification tree for this subsample data set and the agreement index of this tree is 0.43. (b) The first two components of an MDS of the pairwise distances.

The consolidation of the results from the multiple runs of the IRiS pipeline is carried out as follows (see Figure 3). Extending the notion of estimating the age of the most recent common ancestor (MRCA) by combining that of the non-mixing segments carried by it, we take the average of the distances between samples observed in multiple ARGs corresponding to the different instances of the input staged to the IRiS pipeline. This consolidation results in a matrix of pairwise distances of the samples. This matrix is visualized as multidimensional scaling (MDS) plot as well as a classification tree (see Figures 4 and 5). For the Ensemble method, it is worth pointing out that we observed that the number of iterations *N *= 25 is adequate, and no substantial improvement in the classification is obtained for *N *> 25.

### Experimental Setup

#### IRiS pipeline

We first briefly review the IRiS pipeline [6]. Given the genetic information of the extant units, IRiS constructs the ARG that explains the variations. This reconstructed ARG of the samples is necessarily a subgraph of the true ARG, hence is also called a subARG. IRiS constructs the subARG in two phases. A combinatorial algorithm called the DSR [12] is a model-based approach to detecting recombinations in data (with a guaranteed approximation factor [13]). This is based on allelic patterns and thus extends naturally to multi-allelic STRs. In the first phase DSR is run multiple times with different sets of parameters and a statistical consensus [14] is derived from them to produce a matrix of recombination information called the recomatrix. This encodes the local topology information of only the high confidence recombination events detected in the first phase. The subARG is constructed from the recomatrix in the second phase. IRiS, the software tool that implements both the phases is presented in [6].

The validation of the IRiS parameters had earlier been done using population simulations for the human data [6]. However, there is no accessible simulation system that uses STR events, under bilateral transmission, in plant data. Hence we validate our final results against published literature [5]. The IRiS parameter values that we used were the default ones except for the following three. We used grain sizes 1, peak distance 2, and threshold value of 5. The reader is referred to [6] for a detailed description of the parameters and their default values. Note that the very small number of STR polymorphisms does not lend itself to larger grain size values. We demonstrate that these parameter values yield an overall precision rate of 0.92 and recall rate of 0.93 in the classification when the complete data set was used. See the section *Results and discussion *for details.

#### Age estimation

IRiS estimates the age of the internal nodes in the ARG [6]. Note that this is not straightforward due to the presence of mixing segments in the ARG. The estimation is done by first identifying the non-mixing segments being carried at the node and then estimating the depth of the node at each of the non-mixing segment, based on the number of samples carrying the material at that segment. This age is used to compute the pairwise distance of the leaf nodes or the samples.

#### Assessing the results

Let *M *be a 10 × 10 contingency table where each row and each column label is a Cacao genotype as defined in [5]. Thus the [*i*,*j*] of M is an index that compares the population represented by row *i *in [5] to the population represented by column *j *by the ARG method. Next we quantify the agreement seen in *M *by using a suitable index. For this we use the **F-index **[15]. It combines both precision and recall in order to evaluate the agreement of the partitioning solution with respect to the reference clusters.

Each entry of the contingency table *M *is written as *m_i_,_j_*,1 ≤ *i*,*j*≤ 10. *m_i_,_j _*denotes number of elements that belong to the *i*th reference cluster and *j*th computed partition. For each pair *i*, *j*, the **F-index**, *F_i_,_j _*is defined as a weighted harmonic mean of precision (Prec*_i,j_*) and recall (Rec*_i,j_*) values, with weight *α*^2^:

(1) $$F_{i,j}=\frac{1+\alpha^{2}}{1/\text{Pre}{\text{c}}_{i,j}+\alpha^{2}/\text{Re}{\text{c}}_{i,j}}=\frac{\left(\alpha^{2}+1\right)\cdot \text{Pre}{\text{c}}_{i,j}\cdot \text{Re}{\text{c}}_{i,j}}{\alpha^{2}\cdot \text{Pre}{\text{c}}_{i,j}+\text{Re}{\text{c}}_{i,j}},$$

where

$$\text{Pre}{\text{c}}_{i,j}=\frac{m_{i,j}}{p_{j}},\;\text{and}\;\text{Re}{\text{c}}_{i,j}=\frac{m_{i,j}}{c_{i}},\;\text{with}\;p_{j}=\underset{i}{\sum }m_{i,j}\;\text{and}\;c_{i}=\underset{j}{\sum }m_{i,j}.$$

Let $n=\sum_{i}\sum_{j}m_{i,j}$. Here *n *is the total number of samples. Finally, the overall **F-index **for the contingency table is given as [15]:

(2) $$F=\underset{i}{\sum }\left(\frac{c_{i}}{n}\cdot max_{j}\left\{F_{i,j}\right\}\right).$$

Equal weighting for precision and recall is obtained by setting α^2 ^=1.0.

#### Computing the classification trees and the Agreement index

A relaxed version of the NNJ method is used on the pairwise distances of the individuals, derived from the ARG, to obtain the classification tree. The partitions are obtained by pruning the tree and merging the subtrees based on distance until ten clusters are produced. It is worth pointing out that other clustering algorithms could be applied to the distance matrix produced by the solo/esamble method. For instance, when we computed a partition via the Average Linkage and Complete Linkage based on the Ensemble Method, we obtained an **F-index **≈ 0.8 (data shown in Additional File 1). Note that in [5], only the subsample data set has been used to compute the classification tree. Hence, there exists a gold standard in our setup, only for the latter case based on the results available in literature [5].

The partition metric, *d_p _*[16] is used to quantify the agreement between the classification tree *T_1 _*computed by the ARG method and *T_2 _*proposed in [5]:

(3) $$d_{p}\left(T_{1},T_{2}\right)=E\left(T_{1}\right)+E\left(T_{2}\right)-2v\left(T_{1},T_{2}\right),$$

where *E*(*T*) denotes the number of edges of *T *that are not incident on a leaf and *v*(*T*_1_, *T*_2_) denotes the number of identical splits of the taxon induced by deleting an internal edge from each *T*_1 _and *T*_2_. Note that, by definition, 0 ≤ *d_p_*(*T*_1_,*T*_2_) ≤ *E*(*T*_1_) + *E*(*T*_2_). To make this value comparable across different trees, we define an *agreement index *based on Eqn 3 as follows:

(4) $$A=1-\frac{d_{p}\left(T_{1},T_{2}\right)}{E\left(T_{1}\right)+E\left(T_{2}\right)}.$$

In the classification trees some cultivars are split in two or more subtrees. Hence in order to compute the agreement between the two classifications we consider only the class with larger number of samples in order to compute *d_p _*and ignore the smaller subsets.

## Results and discussion

### Classification results

IRiS outputs an ARG for each run. The distance between two samples (extant units) on the ARG is defined to be the age of the MRCA of the two on the ARG [6]. In practice, different non-mixing segments of the extant samples could have distinct MRCAs, since the structure is a subARG. In such cases, an average of age of the different nodes is computed as an estimate of the "distance" between two samples. The procedure to deduce the labels for the classification is discussed in the following section.

#### Labeling the computed partition

We compare this classification, suggested by the ARGs of our analysis, with that of [5] which we call the reference clusters. These clusters are the following genetic groups of cacao according to [5]: Amelonado, Contamana, Criollo, Curaray, Guiana, Iquitos, Marañon, Nacional, Nanay and Purús. We present our comparison results where we cluster the samples into ten groups. To assign names to our partitions, we compute a one-to-one mapping of our partition to that of the clusters of [5] as follows. We first carry out a pairwise intersection of the reference clusters and our computed partitions and populate a 10 × 10 matrix *M' *with the cardinality of these intersection sets, where the rows represent the reference clusters. Then we permute the columns of *M' *in such a manner that the highest values are along the diagonal of this transformed matrix *M*. Such a configuration is indeed possible and there exists a unique *M *for this data. Indeed, the order of the labels of columns is enforced to be the same as that of the rows, implicitly defining the unique one-to-one mapping of the partitions to the reference clusters. The classification trees with these computed labels are displayed in Figures 4 and 5.

### Assessing the results

**Figure 6:**
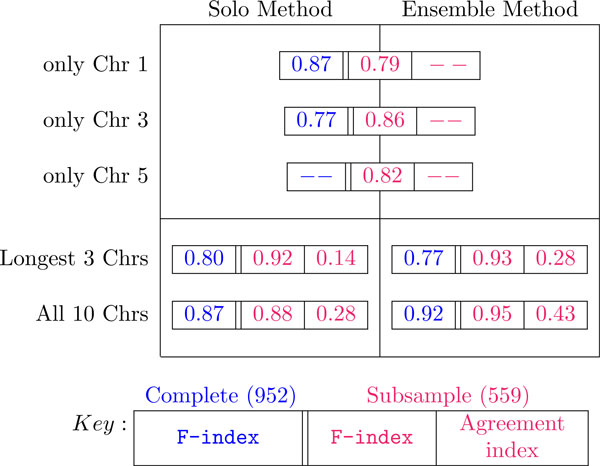
**Summary of the results**. Application of the two methods -Solo and Ensemble- to a variety of data configurations: The results from each case is compared with the gold standard in [5] and the table summarizes the **F-index **and the Agreement metric. The 3 longest chromosomes are Chr 1, Chr 3 and Chr 5. The '-' values are either extremely low or too poor for any classification. The **F-Index **(Eqn 2) and the Agreement index (Eqn 4) are each real positive values between 0.0 and 1.0 inclusive, with the theoretical best at 1.0.

Figure 6 summarizes the results of the two methods on a variety of input configurations. The best results are seen with the ensemble method over all ten chromosomes. Thus more information, in this sparse data set, is indeed better. Using the subsample consistently yields better index values than the complete data set. A detailed discussion follows.

With *α*^2 ^= 1 in Eqn 2 the results are summarized in Tables 1 and 2 for the Ensemble methods on the complete and subsample data set respectively. Note that *F *is an index with value in the range [0,1], and the closer the index is to one the better the agreement between the two partitions. The precision and recall for each of the ten computed partitions are shown in the tables. In particular, an overall precision of 0.91 and a recall of 0.91 is obtained for the complete data set (see Table 1), and an overall precision is 0.93 and a recall of 0.95 is obtained for the subsample data set.

**Table 1:** Contingency table for the ensemble method on all ten chromosomes on the complete data set

	**Ame**.	**Con**.	**Cri**.	**Cur**.	**Gui**.	**Iqu**.	**Mar**.	**Nac**.	**Nan**.	**Pur**.	***c_i_***	**Recall**
			
Amelonado	**68**	1	0	0	6	1	4	0	1	13	94	0.72
Contamana	0	**63**	0	0	0	0	0	3	1	2	69	0.93
Criollo	0	0	**39**	0	0	0	0	0	0	0	39	1.0
Curaray	0	1	0	**114**	0	0	0	2	0	0	117	0.97
Guiana	0	0	0	0	**58**	0	1	0	0	0	59	0.98
Iquitos	0	0	0	0	0	**99**	2	1	6	9	117	0.85
Marañon	2	1	0	0	0	0	**138**	0	0	2	143	0.96
Nacional	2	0	1	2	0	1	0	**46**	0	0	52	0.88
Nanay	0	0	0	0	0	1	0	0	**151**	0	152	0.99
Purùs	0	1	2	1	0	0	0	7	0	**99**	110	0.90
*p_j_*	72	67	42	117	64	102	145	59	159	125	952	
Precision	0.94	0.94	0.93	0.97	0.91	0.97	0.95	0.78	0.95	0.79		

The Ensemble method on all ten chromosomes on the complete data set: The 10 × 10 contingency table *M *for comparing the two partitions is shown above. The reference cluster (*c_i_*) along the rows is from [5]. Our computed partition (*p_j_*) into 10 groups is along the columns. The **F-index **for this matrix is 0.92 (where a perfect agreement has a value of 1). Also the precision $\left(\frac{\text{TP}}{\text{TP}+\text{FP}}\right)$ and recall $\left(\frac{\text{TP}}{\text{TP}+\text{FN}}\right)$ for each computed partition is shown along the last row and the last column respectively.

**Table 2:** Contingency table for the ensemble method on all ten chromosomes on the subsample data set

	**Ame**.	**Con**.	**Cri**.	**Cur**.	**Gui**.	**Iqu**.	**Mar**.	**Nac**.	**Nan**.	**Pur**.	***c_i_***	**Recall**
			
Amelonado	**46**	0	0	0	7	0	0	0	0	0	53	0.86
Contamana	0	**48**	0	0	0	0	0	0	0	0	48	1.0
Criollo	0	0	**27**	0	0	0	0	0	0	0	27	1.0
Curaray	0	1	0	**64**	0	0	0	0	0	0	65	0.98
Guiana	0	0	0	0	**41**	0	0	0	0	0	41	1.0
Iquitos	0	0	0	0	0	**66**	0	0	4	0	70	0.94
Marañon	0	0	5	1	0	0	**95**	1	0	0	102	0.93
Nacional	0	0	0	0	0	0	0	**29**	0	0	29	1.0
Nanay	0	0	0	0	0	0	0	0	**62**	0	62	1.0
Purùs	0	1	0	0	0	6	0	3	0	**52**	62	0.83
*p_j_*	46	50	32	65	48	72	95	33	66	52	559	
Precision	1.0	0.96	0.84	0.98	0.85	0.92	1.0	0.88	0.94	1.0		

The 10 × 10 contingency table *M *in the Ensemble method on all ten chromosomes on the subsample data set. The **F-index **for this matrix is 0.92 (where a perfect agreement has a value of 1).

## Discussion

### Does more data do better ?

Notice that single chromosomes, do not give very good results, nor did aggregating the three longest chromosomes. Hence, even if the number of markers were few on a chromosome, their addition to the data set improved the results across board. However, the subsample data set consistently did better than the complete data set in terms of agreement between their clustering solutions with that one proposed in [5] (more experiments shown in the supplementary material). Thus more markers, but a careful subset of samples, is an optimal choice.

It is interesting to note the two classification trees from the complete and the subsample data set. The two are mostly similar, except for these distinctions. Purús splits almost into two classes in the complete data set and is close to Criollo. However, in the classification tree using the subsample data set, Criollo is closer to Marañon.

### Why does Ensemble Method do better than Solo ?

Cacao, as many other crops, is likely to have been domesticated from a reduced number of individuals carrying a limited amount of variation from the original population. During the process of domestication of wild species, it is reasonable that a strong reduction in the population size and selection of particular traits would cause severe reductions in genetic diversity genome-wide and linkage disequilibrium to increase among loci, maintaining longer haplotypes in the population [17-20]. The bottleneck involved in the domestication process and more importantly the selection of multi-genic traits encompassing long haplotypes from different chromosomes could cause linkage disequilibrium to arise between physically unlinked loci (like those present in different chromosomes). This could explain why the Ensemble Method, that uses the joint information from the whole genome at once to infer the ARG, could recover the demographic structure in cacao more efficiently and accurately than the Solo Method.

### Individual populations

When the complete data set is taken into account (see Table 1), Curaray gives the strongest agreement, with a precision and recall of 0.97. Since, most of the precision and recall results are higher than 0.80 in what follows we discuss only the poorest values. The Amelonado gives a recall of only 0.72 (but a high precision of 0.94), due to the assignment of some samples to the Guiana and Purús cultivars. It is worth pointing out that the Amelonado and Guiana cultivars are very close in the classification trees. Finally, the Nacional cultivars, due to the assignment of few Purús sample, shown a precision value of 0.78 (but a high recall of 0.88). Since the two computational approaches, one based on ARG and the other on the statistics of allelic frequencies, are somewhat orthogonal, it is not immediately clear if this group has been accurately labeled.

When we consider the subsample data set (see Table 2), Amelonado, Marañon and Purús give the strongest agreement, with a precision of 1.0 and recall values greater than 0.80. Criollo and Guiana cultivars shown a precision less then 0.90 (but a high recall of 1.0).

## Conclusions

We demonstrate that IRiS is effective on sparse, multi-allelic, genome-wide STR data. We introduce the ensemble method that naturally extends the ARG model to incorporate multiple chromosomes. This is the first time that a plant population data set is being studied by estimating its underlying ARG. While we have verified the classification of the samples with that in literature, this opens the door to other potential studies that can be made on the ARG. Finally, we corroborate the majority of the groups classified in [5] based on an orthogonal computational approach on the very same data set. We suggest a few candidate groups, whose agreement across the two approaches were not extremely strong, that could benefit from additional molecular markers.

## Competing interests

The authors declare that they have no competing interests.

## Authors' contributions

FU carried out the experiments and LP designed the experiments. DL, OEC, JCM provided the complete data set, and the subsample data set and helped with relevant preprocessing. FU and LP wrote the paper and performed the analysis.

## Supplementary Material

Additional file 1 **Supplementary Material**. The complete set of results for the: (a) the classical clustering algorithms in conjunction with the Euclidean distance; (b) the hiarchical clustering algorithms in conjunction with the distance matrix obtained from the Ensemble method; (c) all the results not included in the main manuscript for the Solo and Ensemble Methods. Click here for file
