# Supplementary material for: ARG-based genome-wide analysis of cacao cultivars
Source: BMC Bioinformatics. 2012 Dec 19;13(Suppl 19):S17. doi: 10.1186/1471-2105-13-S19-S17 (PMC3526434; doi:10.1186/1471-2105-13-S19-S17)
Supplement: Additional file 1 — Supplementary Material. The complete set of results for the: (a) the classical clustering algorithms in conjunction with the Euclidean distance; (b) the hiarchical clustering algorithms in conjunction with the distance matrix obtained from the Ensemble method; (c) all the results not included in the main manuscript for the Solo and Ensemble Methods. [file 1471-2105-13-S19-S17-S1.PDF]

Additional File 1 - ARG-based Genome-wide Analysis of Cacao Cultivars

Filippo Utro[1], Omar E. Cornejo[2], Donald Livingstone[3], Juan Carlos Motamayor[4], and Laxmi Parida[1*]

[1] Computational Biology Center, IBM T. J. Watson Research, Yorktown Heights, NY 10598, USA.
[2] Department of Genetics, Stanford University School of Medicine, Stanford, CA 94305-5120, USA.
[3] USDA, Miami, FL 33186, USA.
[4] Mars Inc., Miami, FL 33158, USA.

Email: futro@us.ibm.com; ocornejo@stanford.edu; donald.livingstone@ars.usda.gov; juan.motamayor@effem.com; parida@us.ibm.com;

*Corresponding author

Outline

In this supplementary, we show the results obtained for the classical clustering algorithms in conjunction with the Euclidean distance. Moreover, based on the results detailed in the main manuscript, we applied the hierarchical clustering algorithms to the distance matrix obtained from the Ensemble method. Finally, all the results not included in the main manuscript for the (1) Solo Method and (2) Ensemble Method are presented here.

Classical Clustering Algorithms

As pointed out in the main manuscript, we use several classical clustering algorithms: K-means, Average Linkage, Complete Linkage, Single Linkage and NNJ. We compute the first four methods producing partitions with a number of clusters from 7 to 15, without obtaining significant results. For brevity, only the cases where the number of clusters equals the one in the gold standard (Motamayor et al) are considered. When the NNJ is computed it was impossible to obtain a useful partition of the data in the desired range, for this reason the results are not provided here. Tables T1-T14 show the results for each algorithms, in conjunction with the Euclidean distance and the iRis pipeline. Since, it is difficult to have a clear map between the clustering solutions of the clustering algorithms with the gold solution, the 10 groups produced by the clustering algorithms are referred to as $G1, G2, \ldots, G10$.

	$G1$	$G2$	$G3$	$G4$	$G5$	$G6$	$G7$	$G8$	$G9$	$G10$	c_i
Amelonado	3	34	42	1	2	2	6	2	1	1	94
Contamana	0	25	23	13	1	2	0	0	5	0	69
Criollo	0	17	8	9	2	0	0	0	3	0	39
Curaray	0	63	22	0	12	7	3	2	2	6	117
Guiana	1	7	34	0	7	0	3	3	0	4	59
Iquitos	0	7	78	0	3	5	4	1	2	17	117
Marañon	2	4	112	0	5	1	5	4	0	10	143
Nacional	3	1	38	0	6	0	2	1	1	0	52
Nanay	3	22	95	1	7	4	6	10	0	4	152
Purùs	1	31	31	0	1	8	26	1	1	10	110
p_j	13	211	483	24	46	27	57	24	15	52	952

Table T1: The 10×10 contingency table M, of the complete data set, for comparing the two partitions: The reference cluster (c_i) along the rows is from Motamyor et al. Our computed partition (p_j) into 10 groups, computed by the K-means clustering algorithms in conjunction with the Euclidean distance, is along the columns. The `F-index` for this matrix is 0.37 (where a perfect agreement has a value of 1).

Euclidean Distance

Complete Data set. With an `F-index` value of 0.37, the best performance is provided by the K-means (see Table T1) where the data is more uniformly distributed. Hoverer, $G2$ and $G3$ contain more than 50% of the individuals. When a hierarchical clustering algorithms is used (see Tables T2-T4), the presence of several singletons induce a poor agreement with the gold standard in Motamayor et al.

Subsample Data set. Again the best performance is obtained by the K-means clustering algorithms with an agreement above the random classification (see Table T5). In particular, the Nanay cultivars are well identified, but all the other cultivars are wrongly classified. For instance, in $G5$ the Amelonado cultivars is identified, but a large number of items from the Purùs cultivar are included in it. In analogy, to the complete data set, when a hierarchical clustering algorithm is used several singletons are identified, see Tables T6-T8.

IRiS pipeline

Given the particular nature of the distance matrix produced by the IRiS pipeline, only the hierarchical clustering algorithms are considered here. Moreover, based on the results discussed in the main manuscript only the Ensemble approach is used.

Complete Data set. The use of IRiS pipelines results in a substantial improvement in the performance of Average Linkage and Complete Linkage, see Tables T9 and T10. Unfortunately the same thing does not happen when the Single Linkage is used (see Table T11). However, it is worth pointing out that the Single

	$G1$	$G2$	$G3$	$G4$	$G5$	$G6$	$G7$	$G8$	$G9$	$G10$	c_i
Amelonado	0	94	0	0	0	0	0	0	0	0	94
Contamana	0	69	0	0	0	0	0	0	0	0	69
Criollo	0	39	0	0	0	0	0	0	0	0	39
Curaray	0	114	1	0	0	0	1	1	0	0	117
Guiana	0	59	0	0	0	0	0	0	0	0	59
Iquitos	0	116	1	0	0	0	0	0	0	0	117
Marañon	0	142	0	0	0	1	0	0	0	0	143
Nacional	0	52	0	0	0	0	0	0	0	0	52
Nanay	0	149	0	1	1	0	0	0	0	1	152
Purùs	1	108	0	0	0	0	0	0	1	0	110
p_j	1	942	2	1	1	1	1	1	1	1	952

Table T2: The 10×10 contingency table M, of the complete data set, for comparing the two partitions: The reference cluster (c_i) along the rows is from Motamyor et al. Our computed partition (p_j) into 10 groups, computed by the Average Linkage clustering algorithms in conjunction with the Euclidean distance, is along the columns. The `F-index` for this matrix is 0.18 (where a perfect agreement has a value of 1).

	$G1$	$G2$	$G3$	$G4$	$G5$	$G6$	$G7$	$G8$	$G9$	$G10$	c_i
Amelonado	0	83	5	2	2	1	0	0	0	1	94
Contamana	1	68	0	0	0	0	0	0	0	0	69
Criollo	1	37	0	0	1	0	0	0	0	0	39
Curaray	1	98	2	1	2	1	0	0	0	12	117
Guiana	3	48	1	0	2	0	0	0	0	7	59
Iquitos	2	103	3	1	0	0	1	0	0	5	117
Marañon	2	129	4	1	0	1	0	0	6	0	143
Nacional	1	42	2	0	0	0	0	0	0	7	52
Nanay	1	133	3	2	0	0	0	1	0	9	152
Purùs	0	85	20	0	1	1	1	0	1	1	110
p_j	12	826	40	7	8	4	2	1	1	51	952

Table T3: The 10×10 contingency table M, of the complete data set, for comparing the two partitions: The reference cluster (c_i) along the rows is from Motamyor et al. Our computed partition (p_j) into 10 groups, computed by the Complete Linkage clustering algorithms in conjunction with the Euclidean distance, is along the columns. The `F-index` for this matrix is 0.19 (where a perfect agreement has a value of 1).

	$G1$	$G2$	$G3$	$G4$	$G5$	$G6$	$G7$	$G8$	$G9$	$G10$	c_i
Amelonado	0	93	1	0	0	0	0	0	0	0	94
Contamana	0	69	0	0	0	0	0	0	0	0	69
Criollo	0	39	0	0	0	0	0	0	0	0	39
Curaray	1	114	0	0	1	0	1	0	0	0	117
Guiana	0	59	0	0	0	0	0	0	0	0	59
Iquitos	0	117	0	0	0	0	0	0	0	0	117
Marañon	0	142	0	1	0	0	0	0	0	0	143
Nacional	0	52	0	0	0	0	0	0	0	0	52
Nanay	0	150	0	0	0	1	0	0	0	1	152
Purùs	0	108	0	0	1	0	0	0	1	0	110
p_j	1	943	1	1	1	1	1	1	1	1	952

Table T4: The 10×10 contingency table M, of the complete data set, for comparing the two partitions: The reference cluster (c_i) along the rows is from Motamyor et al. Our computed partition (p_j) into 10 groups, computed by the Single Linkage clustering algorithms in conjunction with the Euclidean distance, is along the columns. The `F-index` for this matrix is 0.18 (where a perfect agreement has a value of 1).

	$G1$	$G2$	$G3$	$G4$	$G5$	$G6$	$G7$	$G8$	$G9$	$G10$	c_i
Amelonado	0	0	0	0	51	0	0	1	0	1	53
Contamana	0	3	0	0	1	14	0	1	0	29	48
Criollo	0	0	1	0	4	0	0	21	0	1	27
Curaray	0	3	0	0	0	3	0	54	3	0	65
Guiana	0	0	5	3	6	0	0	1	0	0	41
Iquitos	0	4	0	0	0	56	0	0	1	3	70
Marañon	0	3	3	75	19	1	0	0	0	1	102
Nacional	0	1	0	0	1	0	25	0	2	0	29
Nanay	59	0	0	0	1	1	1	0	0	0	62
Purùs	1	0	5	0	33	21	0	1	2	0	62
p_j	60	14	14	78	148	96	26	79	9	35	559

Table T5: The 10×10 contingency table M, of the subsample data set, for comparing the two partitions: The reference cluster (c_i) along the rows is from Motamyor et al. Our computed partition (p_j) into 10 groups, computed by the K-means clustering algorithms in conjunction with the Euclidean distance, is along the columns. The `F-index` for this matrix is 0.65 (where a perfect agreement has a value of 1).

	$G1$	$G2$	$G3$	$G4$	$G5$	$G6$	$G7$	$G8$	$G9$	$G10$	c_i
Amelonado	0	51	0	0	0	0	0	1	1	0	53
Contamana	0	38	0	0	3	2	1	2	1	0	48
Criollo	0	25	0	0	0	0	0	1	0	1	27
Curaray	0	59	4	0	0	0	0	0	2	0	65
Guiana	0	38	0	3	0	0	0	0	0	0	41
Iquitos	0	64	1	0	0	0	0	3	2	0	70
Marañon	1	97	0	0	1	1	1	1	0	0	102
Nacional	0	27	2	0	0	0	0	0	0	0	29
Nanay	0	62	0	0	0	0	0	0	0	0	62
Purùs	0	57	1	0	2	0	0	0	2	0	62
p_j	1	518	8	3	6	3	1	9	9	1	559

Table T6: The 10×10 contingency table M, of the subsample data set, for comparing the two partitions: The reference cluster (c_i) along the rows is from Motamyor et al. Our computed partition (p_j) into 10 groups, computed by the Average Linkage clustering algorithms in conjunction with the Euclidean distance, is along the columns. The `F-index` for this matrix is 0.21 (where a perfect agreement has a value of 1).

	$G1$	$G2$	$G3$	$G4$	$G5$	$G6$	$G7$	$G8$	$G9$	$G10$	c_i
Amelonado	49	1	1	0	0	1	0	0	1	0	53
Contamana	0	1	37	0	1	3	2	3	1	0	48
Criollo	0	24	0	1	0	1	0	0	0	1	27
Curaray	0	0	58	5	0	0	0	0	2	0	65
Guiana	1	34	3	0	0	0	0	3	0	0	41
Iquitos	0	6	56	3	0	3	0	0	2	0	70
Marañon	0	22	74	2	0	1	1	1	1	0	102
Nacional	0	0	26	3	0	0	0	0	0	0	29
Nanay	1	0	60	1	0	0	0	0	0	0	62
Purùs	0	36	21	1	0	0	0	2	2	0	62
p_j	51	124	336	16	1	9	3	9	9	1	559

Table T7: The 10×10 contingency table M, of the subsample data set, for comparing the two partitions: The reference cluster (c_i) along the rows is from Motamyor et al. Our computed partition (p_j) into 10 groups, computed by the Complete Linkage clustering algorithms in conjunction with the Euclidean distance, is along the columns. The `F-index` for this matrix is 0.32 (where a perfect agreement has a value of 1).

	$G1$	$G2$	$G3$	$G4$	$G5$	$G6$	$G7$	$G8$	$G9$	$G10$	c_i
Amelonado	0	0	0	0	0	1	1	51	0	0	53
Contamana	0	1	0	0	3	0	0	43	1	0	48
Criollo	0	0	0	1	0	0	0	25	0	1	27
Curaray	0	2	0	0	0	0	0	63	0	0	65
Guiana	0	0	0	0	0	0	0	41	0	0	41
Iquitos	1	1	0	0	2	0	1	65	0	0	70
Marañon	0	1	0	0	1	0	0	100	0	0	102
Nacional	0	0	0	0	0	0	0	29	0	0	29
Nanay	0	0	0	0	0	0	0	62	0	0	62
Purùs	0	0	2	0	0	0	0	60	0	0	62
p_j	1	5	2	1	6	1	2	539	1	1	559

Table T8: The 10×10 contingency table M, of the subsample data set, for comparing the two partitions: The reference cluster (c_i) along the rows is from Motamyor et al. Our computed partition (p_j) into 10 groups, computed by the Single Linkage clustering algorithms in conjunction with the Euclidean distance, is along the columns. The `F-index` for this matrix is 0.21 (where a perfect agreement has a value of 1).

Linkage shows discouraging results in several analysis see [8-10] and references therein.

Subsample Data set. In analogy to the complete data set, a substantial improvement is obtained for Average and Complete Linkage (see Tables T12 and T13), and a minor improvement is obtained for the Single Linkage (see Table T14). In particular, for the first two algorithms, several cultivars are well identified (e.g. Amelonado, Contamana, Nacional and Nanay).

NNJ

In this section, are detailed the results for different set-up of the Solo/Ensamble method when the relaxed version of the NNJ is used.

Solo

Complete Data set. Table T15 shows the results on the complete data set when all 10 chromosomes are taken into account, and the corresponding MDS and classification trees are shown in Figs. S1 and S2. While, Table T16 and Figs. S3 and S4 show the results when only the first three longest chromosomes are taken into account. Finally, Tables T17 and T18 show the results when only chromosome 1 and 3 are considered. The result when only chromosome 5 is taken into account is not reported, since it is too poor and does not provide any useful indication. In all the mentioned tables, all the groups in each of the respective classification has a high value of Precision or Recall.

	$G1$	$G2$	$G3$	$G4$	$G5$	$G6$	$G7$	$G8$	$G9$	$G10$	c_i
Amelonado	0	2	0	0	73	15	3	0	0	1	94
Contamana	1	0	0	0	0	0	68	0	0	0	69
Criollo	0	0	0	0	0	0	39	0	0	0	39
Curaray	0	1	0	0	0	0	116	0	0	0	117
Guiana	0	0	0	0	0	59	0	0	0	0	59
Iquitos	1	75	4	6	0	2	6	0	22	1	117
Marañon	0	0	0	0	5	102	1	35	0	0	143
Nacional	0	0	0	0	0	0	52	0	0	0	52
Nanay	0	0	1	151	0	0	0	0	0	0	152
Purùs	1	0	0	0	0	0	96	0	13	0	110
p_j	3	78	5	157	78	178	381	35	35	2	952

Table T9: The 10×10 contingency table M, of the complete data set, for comparing the two partitions: The reference cluster (c_i) along the rows is from Motamyor et al. Our computed partition (p_j) into 10 groups, computed by the Average Linkage clustering algorithms in conjunction with the iRis pipeline, is along the columns. The `F-index` for this matrix is 0.59 (where a perfect agreement has a value of 1).

	$G1$	$G2$	$G3$	$G4$	$G5$	$G6$	$G7$	$G8$	$G9$	$G10$	c_i
Amelonado	3	0	5	1	0	0	72	0	13	0	94
Contamana	1	2	0	0	0	0	0	3	0	63	69
Criollo	0	0	0	0	0	0	0	39	0	0	39
Curaray	7	0	0	0	0	0	0	0	0	110	117
Guiana	0	0	0	0	0	0	1	0	58	0	59
Iquitos	6	18	20	5	0	7	3	0	2	0	117
Marañon	1	1	0	0	56	0	2	0	137	2	143
Nacional	36	1	0	0	0	0	0	0	0	15	52
Nanay	0	0	0	1	0	151	0	0	0	0	152
Purùs	3	2	12	20	0	0	0	58	0	15	110
p_j	57	24	37	27	56	158	78	100	210	205	952

Table T10: The 10×10 contingency table M, of the complete data set, for comparing the two partitions: The reference cluster (c_i) along the rows is from Motamyor et al. Our computed partition (p_j) into 10 groups, computed by the Complete Linkage clustering algorithms in conjunction with the iRis pipeline, is along the columns. The `F-index` for this matrix is 0.69 (where a perfect agreement has a value of 1).

	$G1$	$G2$	$G3$	$G4$	$G5$	$G6$	$G7$	$G8$	$G9$	$G10$	c_i
Amelonado	1	87	1	0	1	0	1	1	1	1	94
Contamana	0	69	0	0	0	0	0	0	0	0	69
Criollo	0	39	0	0	0	0	0	0	0	0	39
Curaray	0	116	0	0	0	1	0	0	0	0	117
Guiana	0	59	0	0	0	0	0	0	0	0	59
Iquitos	0	115	0	2	0	0	0	0	0	0	117
Marañon	0	143	0	0	0	0	0	0	0	0	143
Nacional	0	52	0	0	0	0	0	0	0	0	52
Nanay	0	152	0	0	0	0	0	0	0	0	152
Purùs	0	110	0	0	0	0	0	0	0	0	110
p_j	1	942	1	2	1	1	1	1	1	1	952

Table T11: The 10×10 contingency table M, of the complete data set, for comparing the two partitions: The reference cluster (c_i) along the rows is from Motamyor et al. Our computed partition (p_j) into 10 groups, computed by the Single Linkage clustering algorithms in conjunction with the iRis pipeline, is along the columns. The `F-index` for this matrix is 0.19 (where a perfect agreement has a value of 1).

	$G1$	$G2$	$G3$	$G4$	$G5$	$G6$	$G7$	$G8$	$G9$	$G10$	c_i
Amelonado	0	0	8	0	0	1	44	0	0	0	53
Contamana	43	0	0	0	0	5	0	0	0	0	48
Criollo	0	0	0	0	0	27	0	0	0	0	27
Curaray	0	1	0	0	1	63	0	0	0	0	65
Guiana	0	0	0	0	0	0	0	0	41	0	41
Iquitos	0	1	55	13	2	1	0	0	0	0	70
Marañon	0	0	0	0	0	100	0	0	0	0	102
Nacional	0	29	0	0	0	0	0	0	0	0	29
Nanay	0	0	0	0	0	0	0	0	0	62	62
Purùs	1	0	0	5	47	7	0	2	0	0	62
p_j	44	31	63	18	50	204	44	2	41	62	559

Table T12: The 10×10 contingency table M, of the subsample data set, for comparing the two partitions: The reference cluster (c_i) along the rows is from Motamyor et al. Our computed partition (p_j) into 10 groups, computed by the Average Linkage clustering algorithms in conjunction with the iRis pipeline, is along the columns. The `F-index` for this matrix is 0.78 (where a perfect agreement has a value of 1).

	$G1$	$G2$	$G3$	$G4$	$G5$	$G6$	$G7$	$G8$	$G9$	$G10$	c_i
Amelonado	0	8	0	42	0	0	0	0	3	0	53
Contamana	0	0	2	40	0	0	2	3	0	1	48
Criollo	0	0	0	0	24	2	0	1	0	0	27
Curaray	0	0	1	0	5	0	54	0	2	3	65
Guiana	41	0	0	0	0	0	0	0	0	0	41
Iquitos	0	66	0	0	0	0	1	0	1	2	70
Marañon	0	0	16	0	4	16	60	0	0	6	102
Nacional	0	0	0	0	0	0	9	0	0	20	29
Nanay	0	0	0	0	0	0	0	0	0	62	62
Purùs	0	2	3	0	0	38	2	0	5	12	62
p_j	41	76	22	82	33	56	68	64	73	44	559

Table T13: The 10×10 contingency table M, of the subsample data set, for comparing the two partitions: The reference cluster (c_i) along the rows is from Motamyor et al. Our computed partition (p_j) into 10 groups, computed by the Complete Linkage clustering algorithms in conjunction with the iRis pipeline, is along the columns. The `F-index` for this matrix is 0.75 (where a perfect agreement has a value of 1).

	$G1$	$G2$	$G3$	$G4$	$G5$	$G6$	$G7$	$G8$	$G9$	$G10$	c_i
Amelonado	0	52	0	0	0	1	0	0	0	0	53
Contamana	0	48	0	0	0	0	0	0	0	0	48
Criollo	0	27	0	0	0	0	0	0	0	0	27
Curaray	0	65	0	0	0	0	0	0	0	0	65
Guiana	0	40	0	0	0	0	0	0	1	0	41
Iquitos	0	69	0	0	0	0	0	1	0	0	70
Marañon	0	102	0	0	0	0	0	0	0	0	102
Nacional	0	29	0	0	0	0	0	0	0	0	29
Nanay	0	0	1	38	21	0	1	0	0	1	62
Purùs	1	61	0	0	0	0	0	0	0	0	62
p_j	1	493	1	38	21	1	1	1	1	1	559

Table T14: The 10×10 contingency table M, of the subsample data set, for comparing the two partitions: The reference cluster (c_i) along the rows is from Motamyor et al. Our computed partition (p_j) into 10 groups, computed by the Single Linkage clustering algorithms in conjunction with the iRis pipeline, is along the columns. The `F-index` for this matrix is 0.30 (where a perfect agreement has a value of 1).

	Ame.	Con.	Cri.	Cur.	Gui.	Iqu.	Mar.	Nac.	Nan.	Pur.	c_i	Recall
Amelonado	**84**	0	3	0	1	0	6	0	0	0	94	0.89
Contamana	1	**48**	2	9	0	0	0	1	1	7	69	0.69
Criollo	0	0	**38**	0	0	0	0	0	0	1	39	0.97
Curaray	0	0	1	**109**	0	0	0	5	2	0	117	0.88
Guiana	2	0	0	0	**56**	0	1	0	0	0	59	0.95
Iquitos	18	2	2	1	0	**71**	3	2	8	10	117	0.60
Marañon	5	1	0	0	0	0	**133**	0	0	4	143	0.93
Nacional	0	2	1	1	0	0	0	**48**	0	0	52	0.92
Nanay	0	0	0	0	0	1	0	0	**151**	0	152	0.99
Purùs	7	1	4	0	0	4	0	2	2	**90**	110	0.81
p_j	117	54	51	120	57	76	143	58	164	112	952	
Precision	0.71	0.88	0.74	0.90	0.98	0.93	0.92	0.82	0.92	0.80		

Table T15: The 10×10 contingency table M, of the Solo method on the complete data set, for comparing the two partitions: The reference cluster (c_i) along the rows is from Motamyor et al. Our computed partition (p_j) into 10 groups is along the columns. The `F-index` for this matrix is 0.87 (where a perfect agreement has a value of 1). Also the precision $\left(\frac{\text{TP}}{\text{TP+FP}}\right)$ and recall $\left(\frac{\text{TP}}{\text{TP+FN}}\right)$ for each computed partition is shown along the last row and the last column respectively.

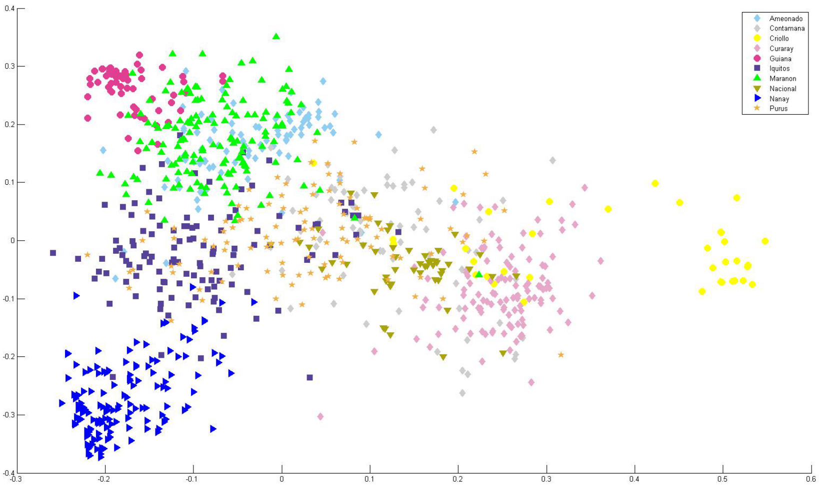

Figure S1: MDS for the Solo method on the complete data set.

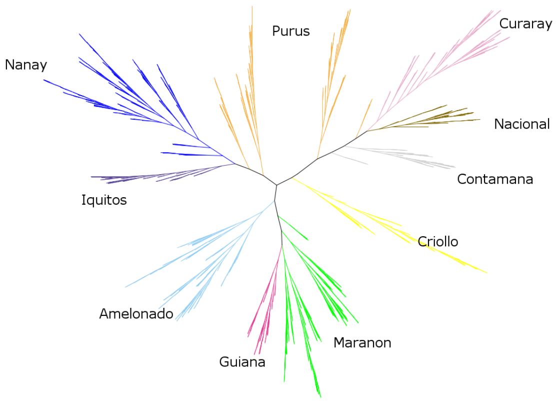

Figure S2: A classification tree of the Solo method on the complete data set.

	Ame.	Con.	Cri.	Cur.	Gui.	Iqu.	Mar.	Nac.	Nan.	Pur.	c_i	Recall
Amelonado	**66**	0	0	2	5	3	10	2	0	6	94	0.70
Contamana	1	**47**	0	2	0	1	3	4	0	11	69	0.68
Criollo	1	0	**35**	0	0	0	0	0	0	3	39	0.89
Curaray	0	1	0	**96**	0	0	0	8	0	12	117	0.82
Guiana	0	0	0	0	**58**	1	0	0	0	0	59	0.98
Iquitos	1	1	0	1	3	**71**	10	4	3	24	117	0.60
Marañon	1	3	0	1	4	9	**112**	0	0	12	143	0.78
Nacional	0	1	0	8	0	0	1	**39**	0	3	52	0.75
Nanay	0	0	0	0	0	0	0	0	**149**	3	152	0.98
Purùs	0	1	0	2	4	2	4	3	0	**94**	110	0.85
p_j	70	54	35	112	74	87	140	60	152	168	952	
Precision	0.94	0.87	1.0	0.86	0.78	0.82	0.80	0.65	0.98	0.56		

Table T16: The 10×10 contingency table M, of the Solo method on the complete data set for the first three longest chromosomes, for comparing the two partitions: The reference cluster (c_i) along the rows is from Motamyor et al. Our computed partition (p_j) into 10 groups is along the columns. The `F-index` for this matrix is 0.80 (where a perfect agreement has a value of 1). Also the precision $\left(\frac{\text{TP}}{\text{TP+FP}}\right)$ and recall $\left(\frac{\text{TP}}{\text{TP+FN}}\right)$ for each computed partition is shown along the last row and the last column respectively.

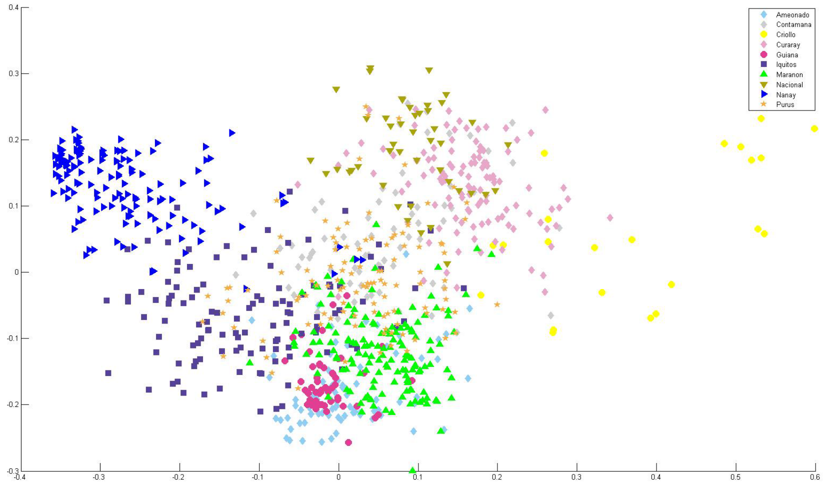

Figure S3: MDS for the Solo method for the first three longest chromosomes on the complete data set.

Subsample Data set. In analogy with the complete data set, all the useful tables and figures for the subsample data set are listed here. Table T19 and Figs. S9 and S10 show the results when all 10 chromosomes are taken into account. Table T20 and Figs. S11 and S12 show the results when only the first three longest chromosomes are taken into account. Finally, the results in Table T21 and Figs. S13-S14 are

	Ame.	Con.	Cri.	Cur.	Gui.	Iqu.	Mar.	Nac.	Nan.	Pur.	c_i	Recall
Amelonado	**77**	2	1	1	3	3	3	0	0	3	94	0.81
Contamana	1	**59**	1	4	0	2	0	1	0	1	69	0.85
Criollo	0	0	**37**	0	0	0	0	0	0	2	39	0.95
Curaray	0	0	1	**112**	0	2	0	0	0	2	117	0.96
Guiana	2	0	0	0	**56**	0	1	0	0	0	59	0.95
Iquitos	1	5	2	3	0	**101**	4	1	0	2	117	0.86
Marañon	11	1	3	0	0	0	**127**	0	0	1	143	0.88
Nacional	0	10	0	4	0	0	0	**38**	0	0	52	0.72
Nanay	0	0	0	0	0	11	0	0	**141**	0	152	0.93
Purùs	0	10	3	6	0	3	2	0	0	**86**	110	0.78
p_j	92	87	48	129	59	122	137	40	141	97	952	
Precision	0.84	0.68	0.77	0.87	0.95	0.89	0.93	0.95	1.0	0.88		

Table T17: The 10×10 contingency table M, for the chromosome 1 on the complete data set, for comparing the two partitions: The reference cluster (c_i) along the rows is from Motamyor et al. Our computed partition (p_j) into 10 groups is along the columns. The `F-index` for this matrix is 0.87 (where a perfect agreement has a value of 1). Also the precision $\left(\frac{\text{TP}}{\text{TP+FP}}\right)$ and recall $\left(\frac{\text{TP}}{\text{TP+FN}}\right)$ for each computed partition is shown along the last row and the last column respectively.

	Ame.	Con.	Cri.	Cur.	Gui.	Iqu.	Mar.	Nac.	Nan.	Pur.	c_i	Recall
Amelonado	**66**	7	0	2	2	2	9	0	6	0	94	0.70
Contamana	12	**33**	4	0	3	0	10	2	5	0	69	0.48
Criollo	0	0	**37**	0	0	0	0	0	0	2	39	0.95
Curaray	0	0	0	**87**	4	16	9	0	0	1	117	0.74
Guiana	0	0	0	0	**56**	0	3	0	0	0	59	0.95
Iquitos	2	0	0	3	7	**93**	0	4	6	3	117	0.79
Marañon	2	0	0	8	3	7	**119**	2	0	1	143	0.83
Nacional	2	0	0	10	0	3	0	**34**	2	1	52	0.65
Nanay	0	0	0	3	0	10	1	1	**136**	1	152	0.89
Purùs	1	1	1	10	6	12	0	5	1	**73**	110	0.66
p_j	85	41	42	123	81	143	151	48	156	82	952	
Precision	0.77	0.80	0.88	0.71	0.69	0.65	0.79	0.71	0.87	0.89		

Table T18: The 10×10 contingency table M, for the chromosome 3 on the complete data set, for comparing the two partitions: The reference cluster (c_i) along the rows is from Motamyor et al. Our computed partition (p_j) into 10 groups is along the columns. The `F-index` for this matrix is 0.77 (where a perfect agreement has a value of 1). Also the precision $\left(\frac{\text{TP}}{\text{TP+FP}}\right)$ and recall $\left(\frac{\text{TP}}{\text{TP+FN}}\right)$ for each computed partition is shown along the last row and the last column respectively.

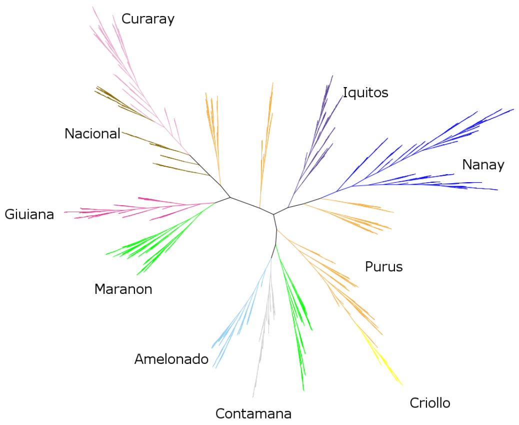

Figure S4: A classification tree of the Solo method for the first three longest chromosomes on the complete data set.

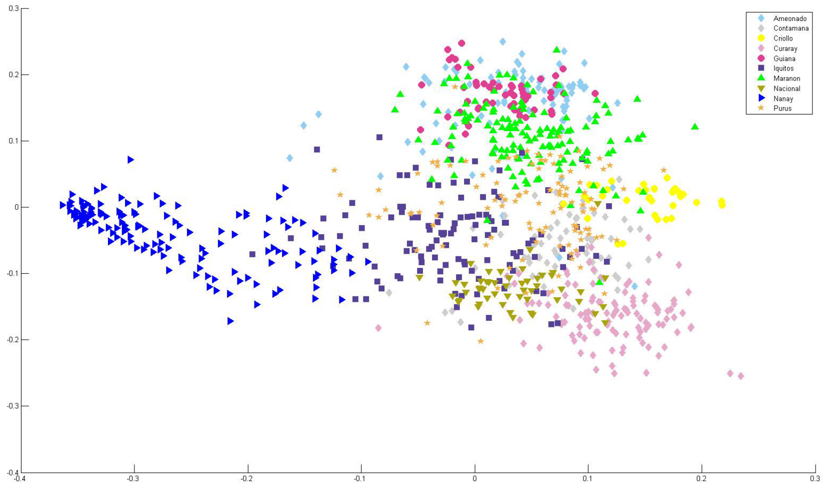

Figure S5: MDS for the Solo method for the chromosome 1 on the complete data set.

for chromosome 1, Table T22 and Figs. S15-S16 are for chromosome 3 and Table T23 and Figs. S17-S18 is for chromosome 5.

	Ame.	Con.	Cri.	Cur.	Gui.	Iqu.	Mar.	Nac.	Nan.	Pur.	c_i	Recall
Amelonado	**49**	1	0	0	0	0	3	0	0	0	53	0.92
Contamana	0	**45**	0	0	0	0	2	1	0	0	48	0.93
Criollo	0	0	**27**	0	0	0	0	0	0	0	27	1.0
Curaray	0	0	0	**63**	0	0	0	2	0	0	65	0.97
Guiana	0	0	0	0	**41**	0	0	0	0	0	41	1.0
Iquitos	1	0	0	0	1	**59**	1	5	0	3	70	0.84
Marañon	0	1	0	0	10	0	**88**	1	0	2	102	0.86
Nacional	0	0	0	0	0	0	0	**29**	0	0	29	1.0
Nanay	0	0	0	0	0	0	0	0	**62**	0	62	1.0
Purùs	1	0	0	0	4	15	9	0	2	**31**	62	0.50
p_j	51	47	27	63	56	74	103	38	64	36	559	
Precision	0.96	0.96	1.0	1.0	0.73	0.79	0.85	0.76	0.97	0.86		

Table T19: The 10×10 contingency table M, for the Solo method on the subsample data set, for comparing the two partitions: The reference cluster (c_i) along the rows is from Motamyor et al. Our computed partition (p_j) into 10 groups is along the columns. The `F-index` for this matrix is 0.88 (where a perfect agreement has a value of 1). Also the precision $\left(\frac{\text{TP}}{\text{TP+FP}}\right)$ and recall $\left(\frac{\text{TP}}{\text{TP+FN}}\right)$ for each computed partition is shown along the last row and the last column respectively.

	Ame.	Con.	Cri.	Cur.	Gui.	Iqu.	Mar.	Nac.	Nan.	Pur.	c_i	Recall
Amelonado	**44**	0	0	1	1	5	2	0	0	0	53	0.83
Contamana	0	**46**	0	1	0	0	0	0	0	1	48	0.96
Criollo	0	0	**27**	0	0	0	0	0	0	0	27	1.0
Curaray	0	0	0	**60**	2	1	0	1	0	1	65	0.92
Guiana	0	0	0	0	**41**	0	0	0	0	0	41	1.0
Iquitos	0	0	0	0	1	**68**	0	0	0	1	70	0.97
Marañon	1	1	0	0	6	1	**92**	0	0	1	102	0.90
Nacional	0	0	0	0	0	0	0	**29**	0	0	29	1.0
Nanay	0	0	0	0	0	0	0	0	**62**	0	62	1.0
Purùs	0	0	0	0	1	4	5	0	0	**52**	62	0.84
p_j	45	47	27	62	52	79	99	30	62	56	559	
Precision	0.98	0.98	1.0	0.97	0.79	0.86	0.93	0.97	1.0	0.93		

Table T20: The 10×10 contingency table M, of the Solo method on the subsample data set for the first three longest chromosomes, for comparing the two partitions: The reference cluster (c_i) along the rows is from Motamyor et al. Our computed partition (p_j) into 10 groups is along the columns. The `F-index` for this matrix is 0.93 (where a perfect agreement has a value of 1). Also the precision $\left(\frac{\text{TP}}{\text{TP+FP}}\right)$ and recall $\left(\frac{\text{TP}}{\text{TP+FN}}\right)$ for each computed partition is shown along the last row and the last column respectively.

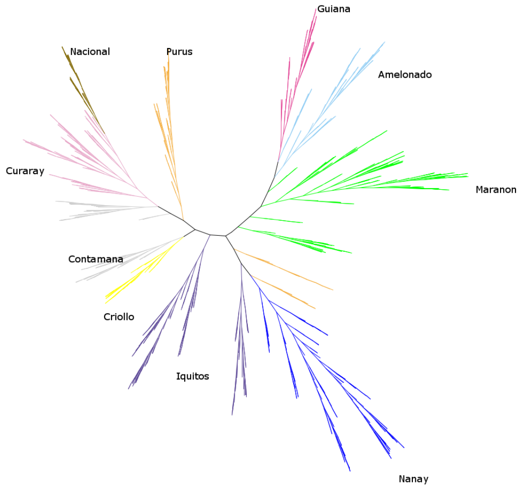

Figure S6: A classification tree of the Solo method for the chromosome 1 on the complete data set.

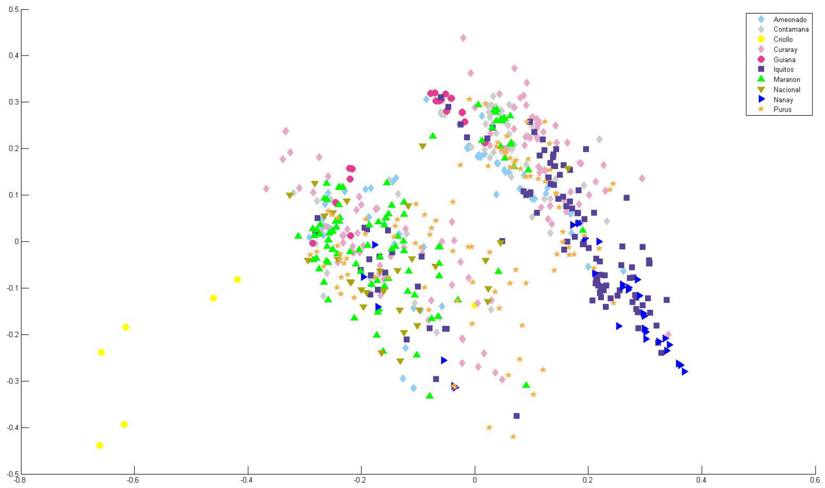

Figure S7: MDS for the Solo method for the chromosome 3 on the complete data set.

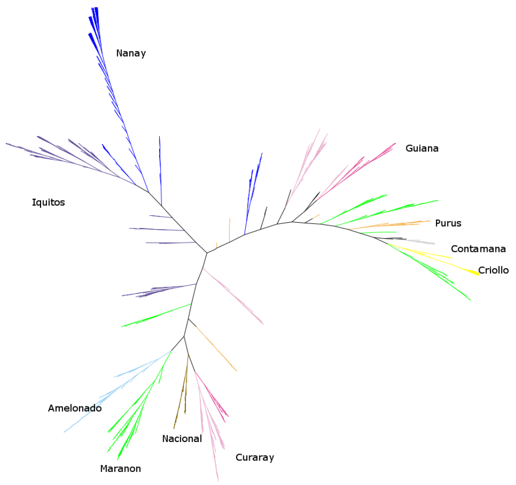

Figure S8: A classification tree of the Solo method for the chromosome 3 on the complete data set.

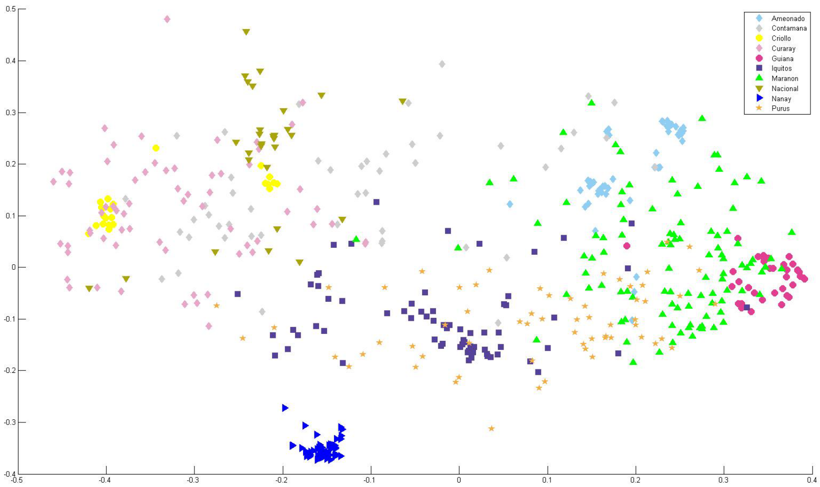

Figure S9: MDS for the Solo method on the subsample data set.

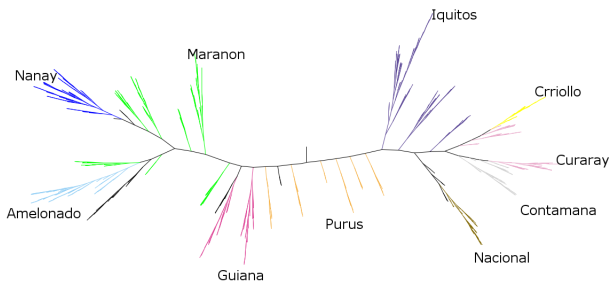

Figure S10: A classification tree of the Solo method on the subsample data set.

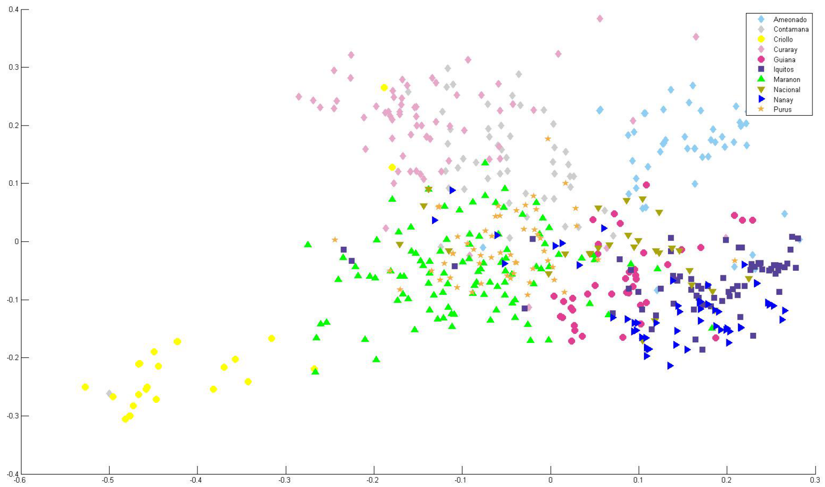

Figure S11: MDS for the Solo method for the first three longest chromosomes on the complete data set.

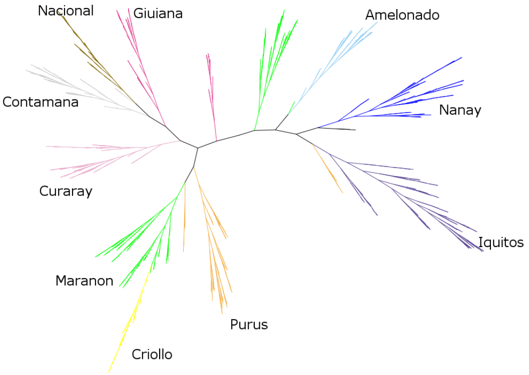

Figure S12: A classification tree of the Solo method for the first three longest chromosomes on the complete data set.

	Ame.	Con.	Cri.	Cur.	Gui.	Iqu.	Mar.	Nac.	Nan.	Pur.	c_i	Recall
Amelonado	**50**	0	0	0	0	0	0	2	1	0	53	0.94
Contamana	9	**21**	2	0	0	5	3	5	0	3	48	0.43
Criollo	0	1	**25**	0	0	0	1	0	0	0	27	0.92
Curaray	4	3	6	**48**	0	0	0	1	0	3	65	0.74
Guiana	0	0	0	1	**33**	0	7	0	0	0	41	0.80
Iquitos	0	0	0	0	0	**56**	0	3	0	11	70	0.80
Marañon	5	6	5	6	0	0	**79**	0	1	0	102	0.77
Nacional	0	0	1	0	0	0	4	**24**	0	0	29	0.82
Nanay	0	0	0	0	0	0	3	0	**59**	0	62	0.95
Purùs	1	3	1	2	0	1	6	0	1	**47**	62	0.75
p_j	69	34	40	57	33	62	103	35	62	64	559	
Precision	0.72	0.62	0.62	0.84	1.0	0.90	0.77	0.69	0.95	0.73		

Table T21: The 10×10 contingency table M, for the chromosome 1 on the complete data set, for comparing the two partitions: The reference cluster (c_i) along the rows is from Motamyor et al. Our computed partition (p_j) into 10 groups is along the columns. The `F-index` for this matrix is 0.79 (where a perfect agreement has a value of 1). Also the precision $\left(\frac{\text{TP}}{\text{TP+FP}}\right)$ and recall $\left(\frac{\text{TP}}{\text{TP+FN}}\right)$ for each computed partition is shown along the last row and the last column respectively.

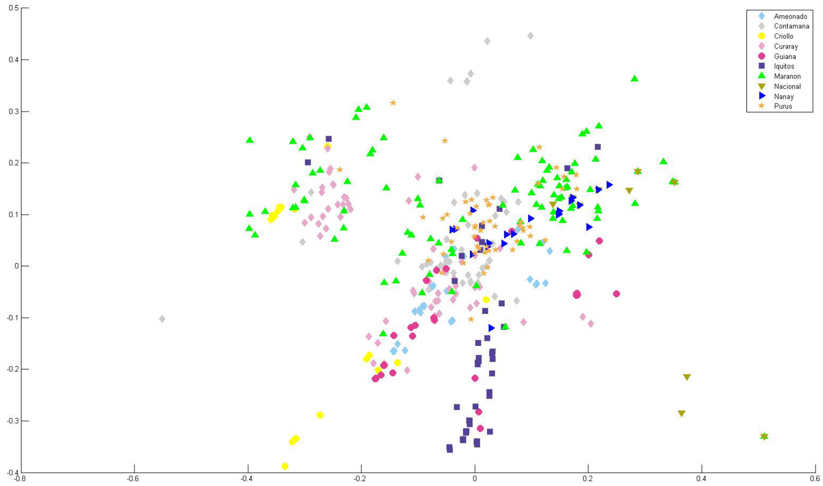

Figure S13: MDS for the Solo method for the chromosome 1 on the subsample data set.

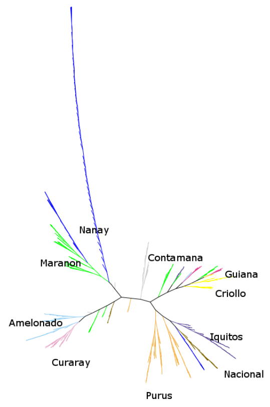

Figure S14: A classification tree of the Solo method for the chromosome 1 on the subsample data set.

	Ame.	Con.	Cri.	Cur.	Gui.	Iqu.	Mar.	Nac.	Nan.	Pur.	c_i	Recall
Amelonado	**46**	1	1	0	0	0	3	0	2	0	53	0.86
Contamana	2	**37**	0	0	0	3	0	0	5	1	48	0.77
Criollo	0	0	**26**	0	0	0	1	0	0	0	27	0.96
Curaray	0	0	0	**57**	1	0	0	0	6	1	65	0.88
Guiana	0	0	0	0	**41**	0	0	0	0	0	41	1.0
Iquitos	2	0	0	0	1	**62**	0	4	1	0	70	0.88
Marañon	0	0	5	0	2	1	**94**	0	0	0	102	0.92
Nacional	0	0	0	0	0	0	4	**23**	0	2	29	0.79
Nanay	1	0	0	0	0	0	1	0	**60**	0	62	0.97
Purùs	0	5	0	0	0	2	11	0	7	**37**	62	0.59
p_j	51	43	32	57	47	68	114	27	81	41	559	
Precision	0.90	0.86	0.81	1.0	0.91	0.91	0.82	0.85	0.74	0.90		

Table T22: The 10×10 contingency table M, for the chromosome 3 on the complete data set, for comparing the two partitions: The reference cluster (c_i) along the rows is from Motamyor et al. Our computed partition (p_j) into 10 groups is along the columns. The `F-index` for this matrix is 0.86 (where a perfect agreement has a value of 1). Also the precision $\left(\frac{\text{TP}}{\text{TP+FP}}\right)$ and recall $\left(\frac{\text{TP}}{\text{TP+FN}}\right)$ for each computed partition is shown along the last row and the last column respectively.

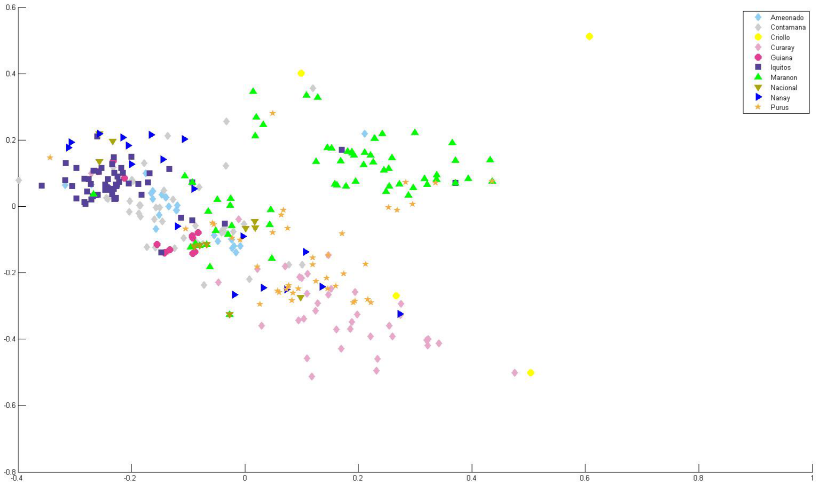

Figure S15: MDS for the Solo method for the chromosome 3 on the subsample data set.

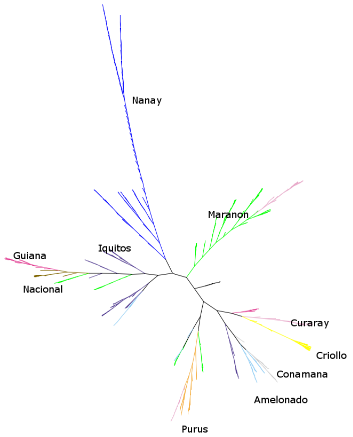

Figure S16: A classification tree of the Solo method for the chromosome 3 on the subsample data set.

	Ame.	Con.	Cri.	Cur.	Gui.	Iqu.	Mar.	Nac.	Nan.	Pur.	c_i	Recall
Amelonado	**45**	0	0	1	0	5	0	0	0	2	53	0.85
Contamana	0	**43**	1	1	0	0	0	2	0	1	48	0.89
Criollo	0	0	**26**	0	0	0	0	0	0	1	27	0.96
Curaray	1	12	0	**48**	0	0	1	3	0	0	65	0.74
Guiana	0	0	0	0	**41**	0	0	0	0	0	41	1.0
Iquitos	5	1	0	0	2	**59**	3	0	0	0	70	0.70
Marañon	5	0	1	5	5	2	**71**	0	0	13	102	0.70
Nacional	0	0	0	2	0	0	1	**26**	0	0	29	0.90
Nanay	0	0	0	0	2	0	0	0	**60**	0	62	0.97
Purùs	0	4	0	1	8	0	6	0	1	**42**	62	0.68
p_j	56	60	28	58	58	66	82	31	61	59	559	
Precision	0.80	0.72	0.93	0.83	0.71	0.89	0.86	0.84	0.96	0.71		

Table T23: The 10×10 contingency table M, for the chromosome 5 on the complete data set, for comparing the two partitions: The reference cluster (c_i) along the rows is from Motamyor et al. Our computed partition (p_j) into 10 groups is along the columns. The `F-index` for this matrix is 0.82 (where a perfect agreement has a value of 1). Also the precision $\left(\frac{\text{TP}}{\text{TP+FP}}\right)$ and recall $\left(\frac{\text{TP}}{\text{TP+FN}}\right)$ for each computed partition is shown along the last row and the last column respectively.

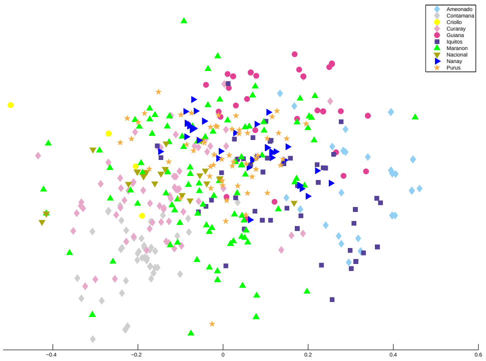

Figure S17: MDS for the Solo method for the chromosome 5 on the subsample data set.

Ensemble

Complete Data set. Table T24 and Figs. S19-S20 show the results only for the first three longest chromosomes.

Subsample Data set. Table T25 and Figs. S21-S22 show the results for only the first three longest chromosomes.

	Ame.	Con.	Cri.	Cur.	Gui.	Iqu.	Mar.	Nac.	Nan.	Pur.	c_i	Recall
Amelonado	**64**	2	0	2	2	3	20	0	0	1	94	0.68
Contamana	0	**43**	3	18	0	0	4	1	0	0	69	0.62
Criollo	0	1	**31**	7	0	0	0	0	0	0	39	0.79
Curaray	0	5	0	**108**	0	0	1	0	0	3	117	0.92
Guiana	0	0	0	0	**52**	0	7	0	0	0	59	0.88
Iquitos	0	7	2	7	3	**76**	20	0	0	3	117	0.65
Marañon	3	10	3	1	1	1	**118**	0	0	5	143	0.82
Nacional	0	1	0	4	0	1	8	**38**	0	0	52	0.73
Nanay	0	0	0	0	0	11	0	0	**141**	0	152	0.93
Purùs	0	13	4	9	4	5	7	0	0	**68**	110	0.62
p_j	67	82	43	156	62	97	185	39	141	80	952	
Precision	0.85	0.52	0.72	0.69	0.84	0.78	0.64	0.97	1.0	0.85		

Table T24: The 10×10 contingency table M, of the Ensemble method on the complete data set for the first three longest chromosomes, for comparing the two partitions: The reference cluster (c_i) along the rows is from Motamyor et al. Our computed partition (p_j) into 10 groups is along the columns. The `F-index` for this matrix is 0.77 (where a perfect agreement has a value of 1). Also the precision $\left(\frac{\text{TP}}{\text{TP+FP}}\right)$ and recall $\left(\frac{\text{TP}}{\text{TP+FN}}\right)$ for each computed partition is shown along the last row and the last column respectively.

	Ame.	Con.	Cri.	Cur.	Gui.	Iqu.	Mar.	Nac.	Nan.	Pur.	c_i	Recall
Amelonado	**44**	0	0	1	0	7	1	0	0	0	53	0.83
Contamana	0	**45**	0	1	0	0	1	1	0	0	48	0.94
Criollo	0	1	**25**	0	0	0	0	1	0	0	27	0.93
Curaray	0	0	0	**57**	0	0	2	3	0	3	65	0.88
Guiana	0	0	0	0	**40**	0	1	0	0	0	41	0.98
Iquitos	0	0	0	1	0	**64**	2	0	0	3	70	0.91
Marañon	0	0	3	0	0	0	**99**	0	0	0	102	0.97
Nacional	0	0	0	0	0	0	0	**23**	0	6	29	0.82
Nanay	0	0	0	0	0	0	0	0	**62**	0	62	1.0
Purùs	0	1	0	3	0	0	2	0	0	**56**	62	0.90
p_j	44	47	28	63	40	71	108	28	62	68	559	
Precision	1.0	0.96	0.89	0.90	1.0	0.90	0.92	0.82	1.0	0.82		

Table T25: The 10×10 contingency table M, of the Ensemble method on the subsample data set for the first three longest chromosomes, for comparing the two partitions: The reference cluster (c_i) along the rows is from Motamyor et al. Our computed partition (p_j) into 10 groups is along the columns. The `F-index` for this matrix is 0.92 (where a perfect agreement has a value of 1). Also the precision $\left(\frac{\text{TP}}{\text{TP+FP}}\right)$ and recall $\left(\frac{\text{TP}}{\text{TP+FN}}\right)$ for each computed partition is shown along the last row and the last column respectively.

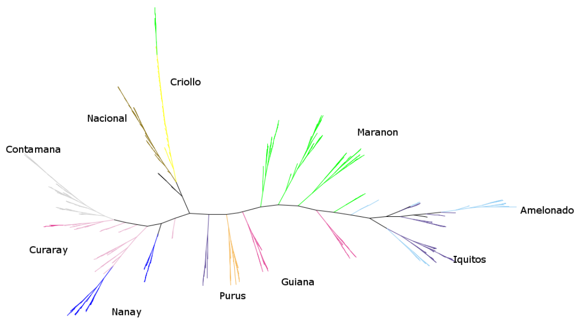

Figure S18: A classification tree of the Solo method for the chromosome 5 on the subsample data set.

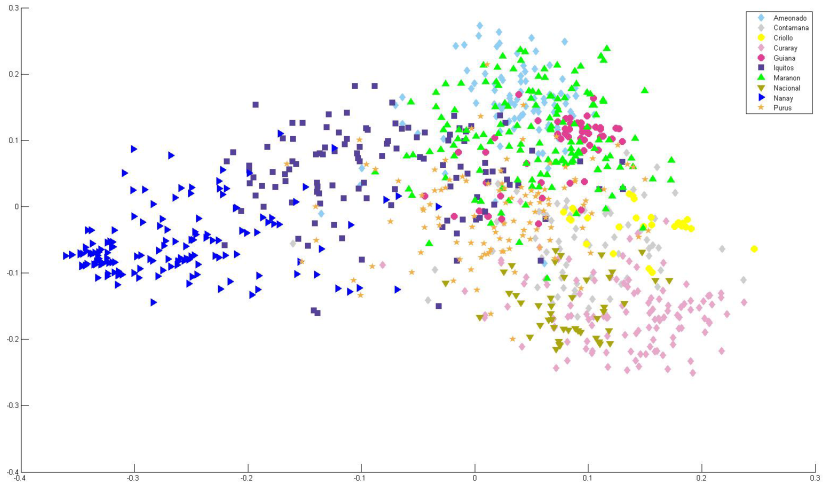

Figure S19: MDS for the Ensemble method for the first three longest chromosomes on the complete data set.

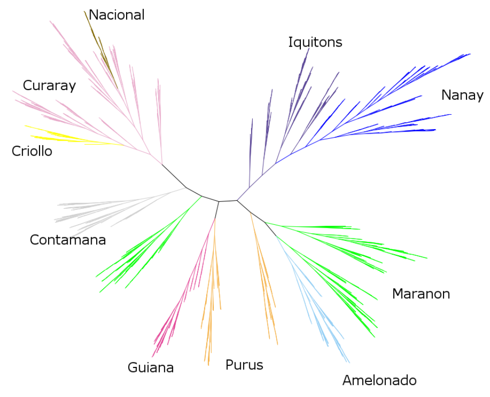

Figure S20: A classification tree of the Ensemble method for the first three longest chromosomes on the complete data set.

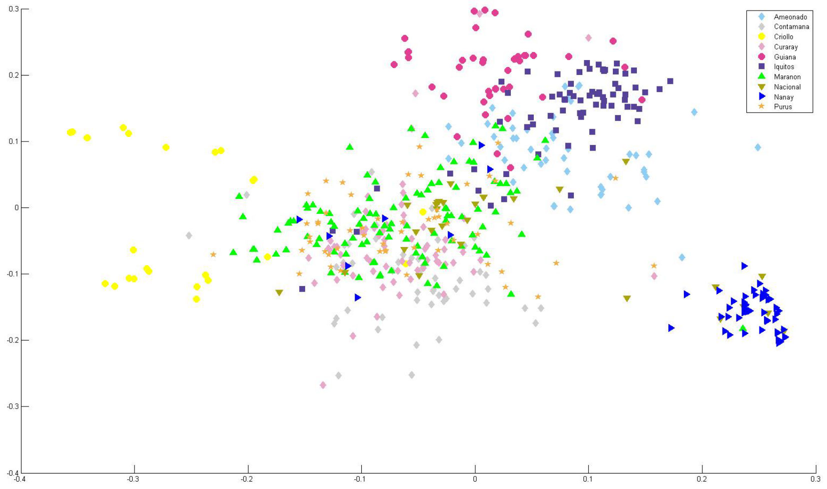

Figure S21: MDS for the Ensemble method for the first three longest chromosomes on the sumbsample data set.

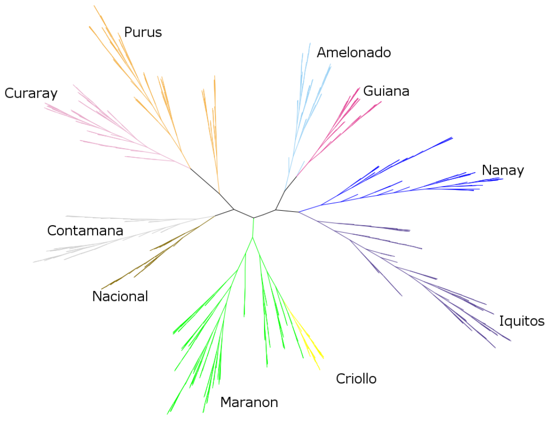

Figure S22: A classification tree of the Ensemble method for the first three longest chromosomes on the sumbsample data set.
